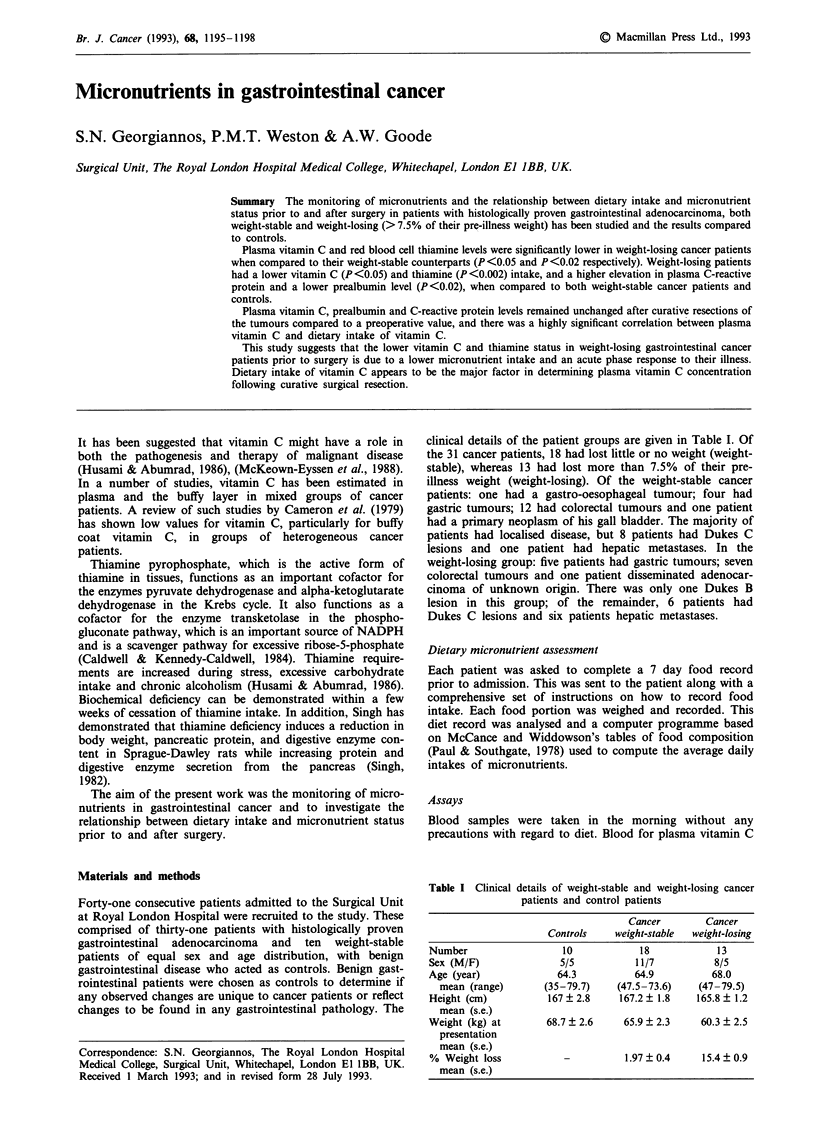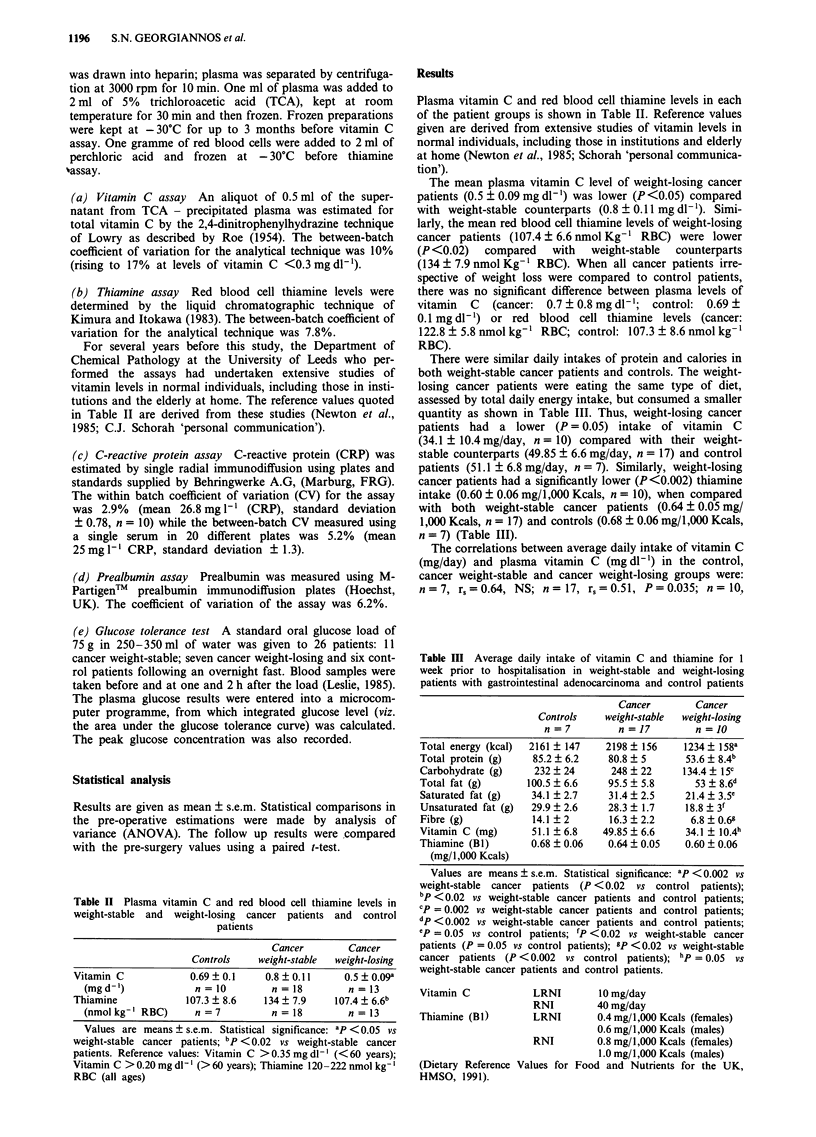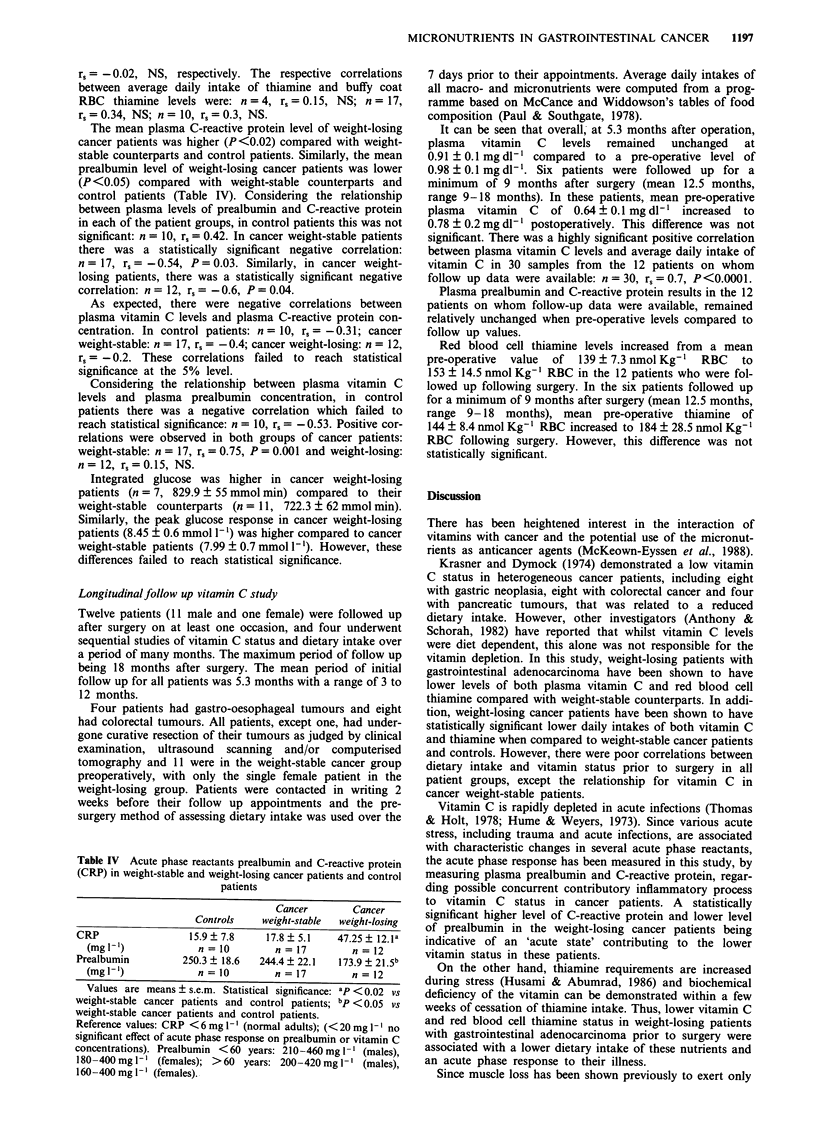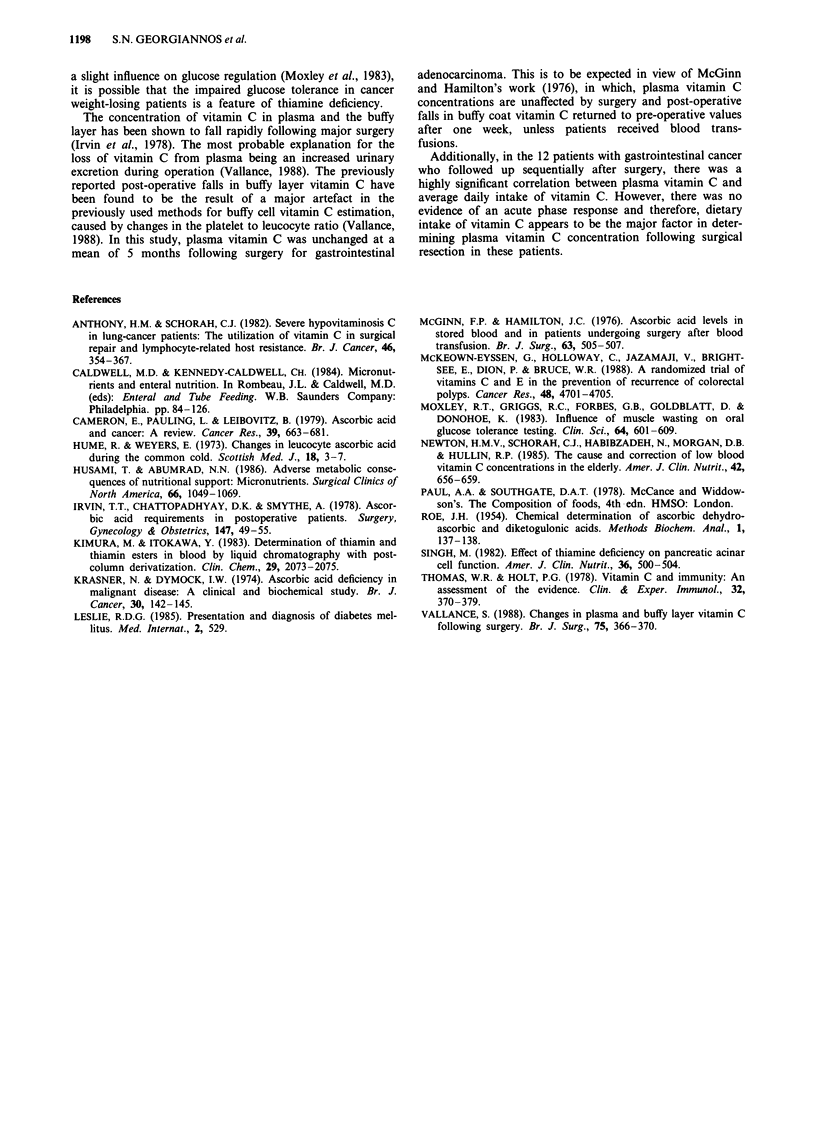# Micronutrients in gastrointestinal cancer.

**DOI:** 10.1038/bjc.1993.503

**Published:** 1993-12

**Authors:** S. N. Georgiannos, P. M. Weston, A. W. Goode

**Affiliations:** Surgical Unit, Royal London Hospital Medical College, Whitechapel, London, U.K.

## Abstract

The monitoring of micronutrients and the relationship between dietary intake and micronutrient status prior to and after surgery in patients with histologically proven gastrointestinal adenocarcinoma, both weight-stable and weight-losing (> 7.5% of their pre-illness weight) has been studied and the results compared to controls. Plasma vitamin C and red blood cell thiamine levels were significantly lower in weight-losing cancer patients when compared to their weight-stable counterparts (P < 0.05 and P < 0.02 respectively). Weight-losing patients had a lower vitamin C (P < 0.05) and thiamine (P < 0.002) intake, and a higher elevation in plasma C-reactive protein and a lower prealbumin level (P < 0.02), when compared to both weight-stable cancer patients and controls. Plasma vitamin C, prealbumin and C-reactive protein levels remained unchanged after curative resections of the tumours compared to a preoperative value, and there was a highly significant correlation between plasma vitamin C and dietary intake of vitamin C. This study suggests that the lower vitamin C and thiamine status in weight-losing gastrointestinal cancer patients prior to surgery is due to a lower micronutrient intake and an acute phase response to their illness. Dietary intake of vitamin C appears to be the major factor in determining plasma vitamin C concentration following curative surgical resection.


Br. J. Cancer (1993), 68, 1195–1198 © Macmillan Press Ltd., 1993

### Micronutrients in gastrointestinal cancer

S.N. Georgiannos, P.M.T. Weston & A.W. Goode

*Surgical Unit, The Royal London Hospital Medical College, Whitechapel, London E1 1BB, UK.*

**Summary** The monitoring of micronutrients and the relationship between dietary intake and micronutrient status prior to and after surgery in patients with histologically proven gastrointestinal adenocarcinoma, both weight-stable and weight-losing (>7.5% of their pre-illness weight) has been studied and the results compared to controls.

Plasma vitamin C and red blood cell thiamine levels were significantly lower in weight-losing cancer patients when compared to their weight-stable counterparts ($P<0.05$ and $P<0.02$ respectively). Weight-losing patients had a lower vitamin C ($P<0.05$) and thiamine ($P<0.002$) intake, and a higher elevation in plasma C-reactive protein and a lower prealbumin level ($P<0.02$), when compared to both weight-stable cancer patients and controls.

Plasma vitamin C, prealbumin and C-reactive protein levels remained unchanged after curative resections of the tumours compared to a preoperative value, and there was a highly significant correlation between plasma vitamin C and dietary intake of vitamin C.

This study suggests that the lower vitamin C and thiamine status in weight-losing gastrointestinal cancer patients prior to surgery is due to a lower micronutrient intake and an acute phase response to their illness. Dietary intake of vitamin C appears to be the major factor in determining plasma vitamin C concentration following curative surgical resection.

It has been suggested that vitamin C might have a role in both the pathogenesis and therapy of malignant disease (Husami & Abumrad, 1986), (McKeown-Eyssen *et al.*, 1988). In a number of studies, vitamin C has been estimated in plasma and the buffy layer in mixed groups of cancer patients. A review of such studies by Cameron *et al.* (1979) has shown low values for vitamin C, particularly for buffy coat vitamin C, in groups of heterogeneous cancer patients.

Thiamine pyrophosphate, which is the active form of thiamine in tissues, functions as an important cofactor for the enzymes pyruvate dehydrogenase and alpha-ketoglutarate dehydrogenase in the Krebs cycle. It also functions as a cofactor for the enzyme transketolase in the phosphogluconate pathway, which is an important source of NADPH and is a scavenger pathway for excessive ribose-5-phosphate (Caldwell & Kennedy-Caldwell, 1984). Thiamine requirements are increased during stress, excessive carbohydrate intake and chronic alcoholism (Husami & Abumrad, 1986). Biochemical deficiency can be demonstrated within a few weeks of cessation of thiamine intake. In addition, Singh has demonstrated that thiamine deficiency induces a reduction in body weight, pancreatic protein, and digestive enzyme content in Sprague-Dawley rats while increasing protein and digestive enzyme secretion from the pancreas (Singh, 1982).

The aim of the present work was the monitoring of micronutrients in gastrointestinal cancer and to investigate the relationship between dietary intake and micronutrient status prior to and after surgery.

### Materials and methods

Forty-one consecutive patients admitted to the Surgical Unit at Royal London Hospital were recruited to the study. These comprised of thirty-one patients with histologically proven gastrointestinal adenocarcinoma and ten weight-stable patients of equal sex and age distribution, with benign gastrointestinal disease who acted as controls. Benign gastrointestinal patients were chosen as controls to determine if any observed changes are unique to cancer patients or reflect changes to be found in any gastrointestinal pathology. The clinical details of the patient groups are given in Table I. Of the 31 cancer patients, 18 had lost little or no weight (weight-stable), whereas 13 had lost more than 7.5% of their pre-illness weight (weight-losing). Of the weight-stable cancer patients: one had a gastro-oesophageal tumour; four had gastric tumours; 12 had colorectal tumours and one patient had a primary neoplasm of his gall bladder. The majority of patients had localised disease, but 8 patients had Dukes C lesions and one patient had hepatic metastases. In the weight-losing group: five patients had gastric tumours; seven colorectal tumours and one patient disseminated adenocarcinoma of unknown origin. There was only one Dukes B lesion in this group; of the remainder, 6 patients had Dukes C lesions and six patients hepatic metastases.

### Dietary micronutrient assessment

Each patient was asked to complete a 7 day food record prior to admission. This was sent to the patient along with a comprehensive set of instructions on how to record food intake. Each food portion was weighed and recorded. This diet record was analysed and a computer programme based on McCance and Widdowson's tables of food composition (Paul & Southgate, 1978) used to compute the average daily intakes of micronutrients.

### Assays

Blood samples were taken in the morning without any precautions with regard to diet. Blood for plasma vitamin C

**Table I** Clinical details of weight-stable and weight-losing cancer patients and control patients

| | *Controls* | *Cancer weight-stable* | *Cancer weight-losing* |
|---|---|---|---|
| Number | 10 | 18 | 13 |
| Sex (M/F) | 5/5 | 11/7 | 8/5 |
| Age (year) mean (range) | 64.3 (35–79.7) | 64.9 (47.5–73.6) | 68.0 (47–79.5) |
| Height (cm) mean (s.e.) | 167 ± 2.8 | 167.2 ± 1.8 | 165.8 ± 1.2 |
| Weight (kg) at presentation mean (s.e.) | 68.7 ± 2.6 | 65.9 ± 2.3 | 60.3 ± 2.5 |
| % Weight loss mean (s.e.) | – | 1.97 ± 0.4 | 15.4 ± 0.9 |

Correspondence: S.N. Georgiannos, The Royal London Hospital Medical College, Surgical Unit, Whitechapel, London E1 1BB, UK.
Received 1 March 1993; and in revised form 28 July 1993.

was drawn into heparin; plasma was separated by centrifugation at 3000 rpm for 10 min. One ml of plasma was added to 2 ml of 5% trichloroacetic acid (TCA), kept at room temperature for 30 min and then frozen. Frozen preparations were kept at −30°C for up to 3 months before vitamin C assay. One gramme of red blood cells were added to 2 ml of perchloric acid and frozen at −30°C before thiamine assay.

*(a) Vitamin C assay* An aliquot of 0.5 ml of the supernatant from TCA – precipitated plasma was estimated for total vitamin C by the 2,4-dinitrophenylhydrazine technique of Lowry as described by Roe (1954). The between-batch coefficient of variation for the analytical technique was 10% (rising to 17% at levels of vitamin C <0.3 mg $dl^{-1}$).

*(b) Thiamine assay* Red blood cell thiamine levels were determined by the liquid chromatographic technique of Kimura and Itokawa (1983). The between-batch coefficient of variation for the analytical technique was 7.8%.

For several years before this study, the Department of Chemical Pathology at the University of Leeds who performed the assays had undertaken extensive studies of vitamin levels in normal individuals, including those in institutions and the elderly at home. The reference values quoted in Table II are derived from these studies (Newton *et al.*, 1985; C.J. Schorah 'personal communication').

*(c) C-reactive protein assay* C-reactive protein (CRP) was estimated by single radial immunodiffusion using plates and standards supplied by Behringwerke A.G, (Marburg, FRG). The within batch coefficient of variation (CV) for the assay was 2.9% (mean 26.8 mg $l^{-1}$ (CRP), standard deviation ± 0.78, $n = 10$) while the between-batch CV measured using a single serum in 20 different plates was 5.2% (mean 25 mg $l^{-1}$ CRP, standard deviation ± 1.3).

*(d) Prealbumin assay* Prealbumin was measured using M-Partigen™ prealbumin immunodiffusion plates (Hoechst, UK). The coefficient of variation of the assay was 6.2%.

*(e) Glucose tolerance test* A standard oral glucose load of 75 g in 250–350 ml of water was given to 26 patients: 11 cancer weight-stable; seven cancer weight-losing and six control patients following an overnight fast. Blood samples were taken before and at one and 2 h after the load (Leslie, 1985). The plasma glucose results were entered into a microcomputer programme, from which integrated glucose level (*viz.* the area under the glucose tolerance curve) was calculated. The peak glucose concentration was also recorded.

### Statistical analysis

Results are given as mean ± s.e.m. Statistical comparisons in the pre-operative estimations were made by analysis of variance (ANOVA). The follow up results were compared with the pre-surgery values using a paired *t*-test.

**Table II** Plasma vitamin C and red blood cell thiamine levels in weight-stable and weight-losing cancer patients and control patients

| | *Controls* | *Cancer weight-stable* | *Cancer weight-losing* |
|---|---|---|---|
| Vitamin C | 0.69 ± 0.1 | 0.8 ± 0.11 | 0.5 ± 0.09[a] |
| (mg $d^{-1}$) | $n = 10$ | $n = 18$ | $n = 13$ |
| Thiamine | 107.3 ± 8.6 | 134 ± 7.9 | 107.4 ± 6.6[b] |
| (nmol $kg^{-1}$ RBC) | $n = 7$ | $n = 18$ | $n = 13$ |

Values are means ± s.e.m. Statistical significance: [a]$P < 0.05$ *vs* weight-stable cancer patients; [b]$P < 0.02$ *vs* weight-stable cancer patients. Reference values: Vitamin C > 0.35 mg $dl^{-1}$ (<60 years); Vitamin C > 0.20 mg $dl^{-1}$ (>60 years); Thiamine 120–222 nmol $kg^{-1}$ RBC (all ages)

### Results

Plasma vitamin C and red blood cell thiamine levels in each of the patient groups is shown in Table II. Reference values given are derived from extensive studies of vitamin levels in normal individuals, including those in institutions and elderly at home (Newton *et al.*, 1985; Schorah 'personal communication').

The mean plasma vitamin C level of weight-losing cancer patients (0.5 ± 0.09 mg $dl^{-1}$) was lower ($P < 0.05$) compared with weight-stable counterparts (0.8 ± 0.11 mg $dl^{-1}$). Similarly, the mean red blood cell thiamine levels of weight-losing cancer patients (107.4 ± 6.6 nmol $Kg^{-1}$ RBC) were lower ($P < 0.02$) compared with weight-stable counterparts (134 ± 7.9 nmol $Kg^{-1}$ RBC). When all cancer patients irrespective of weight loss were compared to control patients, there was no significant difference between plasma levels of vitamin C (cancer: 0.7 ± 0.8 mg $dl^{-1}$; control: 0.69 ± 0.1 mg $dl^{-1}$) or red blood cell thiamine levels (cancer: 122.8 ± 5.8 nmol $kg^{-1}$ RBC; control: 107.3 ± 8.6 nmol $kg^{-1}$ RBC).

There were similar daily intakes of protein and calories in both weight-stable cancer patients and controls. The weight-losing cancer patients were eating the same type of diet, assessed by total daily energy intake, but consumed a smaller quantity as shown in Table III. Thus, weight-losing cancer patients had a lower ($P = 0.05$) intake of vitamin C (34.1 ± 10.4 mg/day, $n = 10$) compared with their weight-stable counterparts (49.85 ± 6.6 mg/day, $n = 17$) and control patients (51.1 ± 6.8 mg/day, $n = 7$). Similarly, weight-losing cancer patients had a significantly lower ($P < 0.002$) thiamine intake (0.60 ± 0.06 mg/1,000 Kcals, $n = 10$), when compared with both weight-stable cancer patients (0.64 ± 0.05 mg/1,000 Kcals, $n = 17$) and controls (0.68 ± 0.06 mg/1,000 Kcals, $n = 7$) (Table III).

The correlations between average daily intake of vitamin C (mg/day) and plasma vitamin C (mg $dl^{-1}$) in the control, cancer weight-stable and cancer weight-losing groups were: $n = 7$, $r_s = 0.64$, NS; $n = 17$, $r_s = 0.51$, $P = 0.035$; $n = 10$,

**Table III** Average daily intake of vitamin C and thiamine for 1 week prior to hospitalisation in weight-stable and weight-losing patients with gastrointestinal adenocarcinoma and control patients

| | *Controls* $n = 7$ | *Cancer weight-stable* $n = 17$ | *Cancer weight-losing* $n = 10$ |
|---|---|---|---|
| Total energy (kcal) | 2161 ± 147 | 2198 ± 156 | 1234 ± 158[a] |
| Total protein (g) | 85.2 ± 6.2 | 80.8 ± 5 | 53.6 ± 8.4[b] |
| Carbohydrate (g) | 232 ± 24 | 248 ± 22 | 134.4 ± 15[c] |
| Total fat (g) | 100.5 ± 6.6 | 95.5 ± 5.8 | 53 ± 8.6[d] |
| Saturated fat (g) | 34.1 ± 2.7 | 31.4 ± 2.5 | 21.4 ± 3.5[e] |
| Unsaturated fat (g) | 29.9 ± 2.6 | 28.3 ± 1.7 | 18.8 ± 3[f] |
| Fibre (g) | 14.1 ± 2 | 16.3 ± 2.2 | 6.8 ± 0.6[g] |
| Vitamin C (mg) | 51.1 ± 6.8 | 49.85 ± 6.6 | 34.1 ± 10.4[h] |
| Thiamine (B1) (mg/1,000 Kcals) | 0.68 ± 0.06 | 0.64 ± 0.05 | 0.60 ± 0.06 |

Values are means ± s.e.m. Statistical significance: [a]$P < 0.002$ *vs* weight-stable cancer patients ($P < 0.02$ *vs* control patients); [b]$P < 0.02$ *vs* weight-stable cancer patients and control patients; [c]$P = 0.002$ *vs* weight-stable cancer patients and control patients; [d]$P < 0.002$ *vs* weight-stable cancer patients and control patients; [e]$P = 0.05$ *vs* control patients; [f]$P < 0.02$ *vs* weight-stable cancer patients ($P = 0.05$ *vs* control patients); [g]$P < 0.02$ *vs* weight-stable cancer patients ($P < 0.002$ *vs* control patients); [h]$P = 0.05$ *vs* weight-stable cancer patients and control patients.

| | | |
|---|---|---|
| Vitamin C | LRNI | 10 mg/day |
| | RNI | 40 mg/day |
| Thiamine (B1) | LRNI | 0.4 mg/1,000 Kcals (females) |
| | | 0.6 mg/1,000 Kcals (males) |
| | RNI | 0.8 mg/1,000 Kcals (females) |
| | | 1.0 mg/1,000 Kcals (males) |

(Dietary Reference Values for Food and Nutrients for the UK, HMSO, 1991).

$r_s = -0.02$, NS, respectively. The respective correlations between average daily intake of thiamine and buffy coat RBC thiamine levels were: $n = 4$, $r_s = 0.15$, NS; $n = 17$, $r_s = 0.34$, NS; $n = 10$, $r_s = 0.3$, NS.

The mean plasma C-reactive protein level of weight-losing cancer patients was higher ($P < 0.02$) compared with weight-stable counterparts and control patients. Similarly, the mean prealbumin level of weight-losing cancer patients was lower ($P < 0.05$) compared with weight-stable counterparts and control patients (Table IV). Considering the relationship between plasma levels of prealbumin and C-reactive protein in each of the patient groups, in control patients this was not significant: $n = 10$, $r_s = 0.42$. In cancer weight-stable patients there was a statistically significant negative correlation: $n = 17$, $r_s = -0.54$, $P = 0.03$. Similarly, in cancer weight-losing patients, there was a statistically significant negative correlation: $n = 12$, $r_s = -0.6$, $P = 0.04$.

As expected, there were negative correlations between plasma vitamin C levels and plasma C-reactive protein concentration. In control patients: $n = 10$, $r_s = -0.31$; cancer weight-stable: $n = 17$, $r_s = -0.4$; cancer weight-losing: $n = 12$, $r_s = -0.2$. These correlations failed to reach statistical significance at the 5% level.

Considering the relationship between plasma vitamin C levels and plasma prealbumin concentration, in control patients there was a negative correlation which failed to reach statistical significance: $n = 10$, $r_s = -0.53$. Positive correlations were observed in both groups of cancer patients: weight-stable: $n = 17$, $r_s = 0.75$, $P = 0.001$ and weight-losing: $n = 12$, $r_s = 0.15$, NS.

Integrated glucose was higher in cancer weight-losing patients ($n = 7$, $829.9 \pm 55$ mmol min) compared to their weight-stable counterparts ($n = 11$, $722.3 \pm 62$ mmol min). Similarly, the peak glucose response in cancer weight-losing patients ($8.45 \pm 0.6$ mmol $l^{-1}$) was higher compared to cancer weight-stable patients ($7.99 \pm 0.7$ mmol $l^{-1}$). However, these differences failed to reach statistical significance.

### *Longitudinal follow up vitamin C study*

Twelve patients (11 male and one female) were followed up after surgery on at least one occasion, and four underwent sequential studies of vitamin C status and dietary intake over a period of many months. The maximum period of follow up being 18 months after surgery. The mean period of initial follow up for all patients was 5.3 months with a range of 3 to 12 months.

Four patients had gastro-oesophageal tumours and eight had colorectal tumours. All patients, except one, had undergone curative resection of their tumours as judged by clinical examination, ultrasound scanning and/or computerised tomography and 11 were in the weight-stable cancer group preoperatively, with only the single female patient in the weight-losing group. Patients were contacted in writing 2 weeks before their follow up appointments and the pre-surgery method of assessing dietary intake was used over the 7 days prior to their appointments. Average daily intakes of all macro- and micronutrients were computed from a programme based on McCance and Widdowson's tables of food composition (Paul & Southgate, 1978).

It can be seen that overall, at 5.3 months after operation, plasma vitamin C levels remained unchanged at $0.91 \pm 0.1$ mg $dl^{-1}$ compared to a pre-operative level of $0.98 \pm 0.1$ mg $dl^{-1}$. Six patients were followed up for a minimum of 9 months after surgery (mean 12.5 months, range 9–18 months). In these patients, mean pre-operative plasma vitamin C of $0.64 \pm 0.1$ mg $dl^{-1}$ increased to $0.78 \pm 0.2$ mg $dl^{-1}$ postoperatively. This difference was not significant. There was a highly significant positive correlation between plasma vitamin C levels and average daily intake of vitamin C in 30 samples from the 12 patients on whom follow up data were available: $n = 30$, $r_s = 0.7$, $P < 0.0001$.

Plasma prealbumin and C-reactive protein results in the 12 patients on whom follow-up data were available, remained relatively unchanged when pre-operative levels compared to follow up values.

Red blood cell thiamine levels increased from a mean pre-operative value of $139 \pm 7.3$ nmol $Kg^{-1}$ RBC to $153 \pm 14.5$ nmol $Kg^{-1}$ RBC in the 12 patients who were followed up following surgery. In the six patients followed up for a minimum of 9 months after surgery (mean 12.5 months, range 9–18 months), mean pre-operative thiamine of $144 \pm 8.4$ nmol $Kg^{-1}$ RBC increased to $184 \pm 28.5$ nmol $Kg^{-1}$ RBC following surgery. However, this difference was not statistically significant.

**Table IV** Acute phase reactants prealbumin and C-reactive protein (CRP) in weight-stable and weight-losing cancer patients and control patients

| | *Controls* | *Cancer weight-stable* | *Cancer weight-losing* |
|---|---|---|---|
| CRP | $15.9 \pm 7.8$ | $17.8 \pm 5.1$ | $47.25 \pm 12.1^a$ |
| (mg $l^{-1}$) | $n = 10$ | $n = 17$ | $n = 12$ |
| Prealbumin | $250.3 \pm 18.6$ | $244.4 \pm 22.1$ | $173.9 \pm 21.5^b$ |
| (mg $l^{-1}$) | $n = 10$ | $n = 17$ | $n = 12$ |

Values are means ± s.e.m. Statistical significance: $^aP < 0.02$ *vs* weight-stable cancer patients and control patients; $^bP < 0.05$ *vs* weight-stable cancer patients and control patients.
Reference values: CRP <6 mg $l^{-1}$ (normal adults); (<20 mg $l^{-1}$ no significant effect of acute phase response on prealbumin or vitamin C concentrations). Prealbumin <60 years: 210–460 mg $l^{-1}$ (males), 180–400 mg $l^{-1}$ (females); >60 years: 200–420 mg $l^{-1}$ (males), 160–400 mg $l^{-1}$ (females).

### Discussion

There has been heightened interest in the interaction of vitamins with cancer and the potential use of the micronutrients as anticancer agents (McKeown-Eyssen *et al.*, 1988).

Krasner and Dymock (1974) demonstrated a low vitamin C status in heterogeneous cancer patients, including eight with gastric neoplasia, eight with colorectal cancer and four with pancreatic tumours, that was related to a reduced dietary intake. However, other investigators (Anthony & Schorah, 1982) have reported that whilst vitamin C levels were diet dependent, this alone was not responsible for the vitamin depletion. In this study, weight-losing patients with gastrointestinal adenocarcinoma have been shown to have lower levels of both plasma vitamin C and red blood cell thiamine compared with weight-stable counterparts. In addition, weight-losing cancer patients have been shown to have statistically significant lower daily intakes of both vitamin C and thiamine when compared to weight-stable cancer patients and controls. However, there were poor correlations between dietary intake and vitamin status prior to surgery in all patient groups, except the relationship for vitamin C in cancer weight-stable patients.

Vitamin C is rapidly depleted in acute infections (Thomas & Holt, 1978; Hume & Weyers, 1973). Since various acute stress, including trauma and acute infections, are associated with characteristic changes in several acute phase reactants, the acute phase response has been measured in this study, by measuring plasma prealbumin and C-reactive protein, regarding possible concurrent contributory inflammatory process to vitamin C status in cancer patients. A statistically significant higher level of C-reactive protein and lower level of prealbumin in the weight-losing cancer patients being indicative of an 'acute state' contributing to the lower vitamin status in these patients.

On the other hand, thiamine requirements are increased during stress (Husami & Abumrad, 1986) and biochemical deficiency of the vitamin can be demonstrated within a few weeks of cessation of thiamine intake. Thus, lower vitamin C and red blood cell thiamine status in weight-losing patients with gastrointestinal adenocarcinoma prior to surgery were associated with a lower dietary intake of these nutrients and an acute phase response to their illness.

Since muscle loss has been shown previously to exert only a slight influence on glucose regulation (Moxley *et al.*, 1983), it is possible that the impaired glucose tolerance in cancer weight-losing patients is a feature of thiamine deficiency.

The concentration of vitamin C in plasma and the buffy layer has been shown to fall rapidly following major surgery (Irvin *et al.*, 1978). The most probable explanation for the loss of vitamin C from plasma being an increased urinary excretion during operation (Vallance, 1988). The previously reported post-operative falls in buffy layer vitamin C have been found to be the result of a major artefact in the previously used methods for buffy cell vitamin C estimation, caused by changes in the platelet to leucocyte ratio (Vallance, 1988). In this study, plasma vitamin C was unchanged at a mean of 5 months following surgery for gastrointestinal adenocarcinoma. This is to be expected in view of McGinn and Hamilton's work (1976), in which, plasma vitamin C concentrations are unaffected by surgery and post-operative falls in buffy coat vitamin C returned to pre-operative values after one week, unless patients received blood transfusions.

Additionally, in the 12 patients with gastrointestinal cancer who followed up sequentially after surgery, there was a highly significant correlation between plasma vitamin C and average daily intake of vitamin C. However, there was no evidence of an acute phase response and therefore, dietary intake of vitamin C appears to be the major factor in determining plasma vitamin C concentration following surgical resection in these patients.

### References

ANTHONY, H.M. & SCHORAH, C.J. (1982). Severe hypovitaminosis C in lung-cancer patients: The utilization of vitamin C in surgical repair and lymphocyte-related host resistance. *Br. J. Cancer*, **46**, 354–367.

CALDWELL, M.D. & KENNEDY-CALDWELL, CH. (1984). Micronutrients and enteral nutrition. In Rombeau, J.L. & Caldwell, M.D. (eds): *Enteral and Tube Feeding*. W.B. Saunders Company: Philadelphia. pp. 84–126.

CAMERON, E., PAULING, L. & LEIBOVITZ, B. (1979). Ascorbic acid and cancer: A review. *Cancer Res.*, **39**, 663–681.

HUME, R. & WEYERS, E. (1973). Changes in leucocyte ascorbic acid during the common cold. *Scottish Med. J.*, **18**, 3–7.

HUSAMI, T. & ABUMRAD, N.N. (1986). Adverse metabolic consequences of nutritional support: Micronutrients. *Surgical Clinics of North America*, **66**, 1049–1069.

IRVIN, T.T., CHATTOPADHYAY, D.K. & SMYTHE, A. (1978). Ascorbic acid requirements in postoperative patients. *Surgery, Gynecology & Obstetrics*, **147**, 49–55.

KIMURA, M. & ITOKAWA, Y. (1983). Determination of thiamin and thiamin esters in blood by liquid chromatography with post-column derivatization. *Clin. Chem.*, **29**, 2073–2075.

KRASNER, N. & DYMOCK, I.W. (1974). Ascorbic acid deficiency in malignant disease: A clinical and biochemical study. *Br. J. Cancer*, **30**, 142–145.

LESLIE, R.D.G. (1985). Presentation and diagnosis of diabetes mellitus. *Med. Internat.*, **2**, 529.

MCGINN, F.P. & HAMILTON, J.C. (1976). Ascorbic acid levels in stored blood and in patients undergoing surgery after blood transfusion. *Br. J. Surg.*, **63**, 505–507.

MCKEOWN-EYSSEN, G., HOLLOWAY, C., JAZAMAJI, V., BRIGHT-SEE, E., DION, P. & BRUCE, W.R. (1988). A randomized trial of vitamins C and E in the prevention of recurrence of colorectal polyps. *Cancer Res.*, **48**, 4701–4705.

MOXLEY, R.T., GRIGGS, R.C., FORBES, G.B., GOLDBLATT, D. & DONOHOE, K. (1983). Influence of muscle wasting on oral glucose tolerance testing. *Clin. Sci.*, **64**, 601–609.

NEWTON, H.M.V., SCHORAH, C.J., HABIBZADEH, N., MORGAN, D.B. & HULLIN, R.P. (1985). The cause and correction of low blood vitamin C concentrations in the elderly. *Amer. J. Clin. Nutrit.*, **42**, 656–659.

PAUL, A.A. & SOUTHGATE, D.A.T. (1978). McCance and Widdowson's. The Composition of foods, 4th edn. HMSO: London.

ROE, J.H. (1954). Chemical determination of ascorbic dehydroascorbic and diketogulonic acids. *Methods Biochem. Anal.*, **1**, 137–138.

SINGH, M. (1982). Effect of thiamine deficiency on pancreatic acinar cell function. *Amer. J. Clin. Nutrit.*, **36**, 500–504.

THOMAS, W.R. & HOLT, P.G. (1978). Vitamin C and immunity: An assessment of the evidence. *Clin. & Exper. Immunol.*, **32**, 370–379.

VALLANCE, S. (1988). Changes in plasma and buffy layer vitamin C following surgery. *Br. J. Surg.*, **75**, 366–370.